# Association of Antenatal Steroid Exposure With Survival Among Infants Receiving Postnatal Life Support at 22 to 25 Weeks’ Gestation

**DOI:** 10.1001/jamanetworkopen.2018.3235

**Published:** 2018-10-12

**Authors:** Danielle E. Y. Ehret, Erika M. Edwards, Lucy T. Greenberg, Ira M. Bernstein, Jeffrey S. Buzas, Roger F. Soll, Jeffrey D. Horbar

**Affiliations:** 1 Vermont Oxford Network, Burlington, Vermont; 2 Department of Pediatrics, Robert Larner College of Medicine, University of Vermont, Burlington; 3 Department of Mathematics and Statistics, College of Engineering and Mathematical Sciences, University of Vermont, Burlington; 4 Department of Obstetrics, Gynecology, and Reproductive Services, Robert Larner College of Medicine, University of Vermont, Burlington

## Abstract

**Importance:**

Although evidence of antenatal steroids (ANS) efficacy at 22 to 25 weeks’ gestation is limited, increasingly these infants are treated with postnatal life support.

**Objectives:**

To estimate the proportion of infants receiving postnatal life support at 22 to 25 weeks’ gestation who had exposure to ANS, and to examine if the provision of ANS was associated with a higher rate of survival to hospital discharge and survival without major morbidities.

**Design, Setting, and Participants:**

This multicenter observational cohort study consisted of 33 472 eligible infants liveborn at 431 US Vermont Oxford Network member hospitals between January 1, 2012, and December 31, 2016. We excluded infants with recognized syndromes or major congenital anomalies. Of the eligible infants, 29 932 received postnatal life support and were included in the analyses. Data analysis was conducted from July 2017 to July 2018.

**Exposure:**

Antenatal steroids administered to the mother at any time prior to delivery.

**Main Outcomes and Measures:**

Survival to hospital discharge, major morbidities among survivors, and the composite of survival to discharge without major morbidities.

**Results:**

Among 29 932 infants who received postnatal life support, 51.9% were male, with a mean (SD) gestational age of 24.12 (0.86) weeks and mean (SD) birth weight of 668 (140) g; 26 090 (87.2%) had ANS exposure and 3842 (12.8%) had no ANS exposure. Survival to hospital discharge was higher for infants with ANS exposure (18 717 of 25 892 [72.3%]) compared with infants without ANS exposure (1981 of 3820 [51.9%]); the adjusted risk ratio for 22 weeks was 2.11 (95% CI, 1.68-2.65), for 23 weeks was 1.54 (95% CI, 1.40-1.70), for 24 weeks was 1.18 (95% CI, 1.12-1.25), and for 25 weeks was 1.11 (95% CI, 1.07-1.14). Survival to hospital discharge without major morbidities was higher for infants with ANS exposure (3777 of 25 833 [14.6%]) compared with infants without ANS exposure (347 of 3806 [9.1%]); the adjusted risk ratio for 22 through 25 weeks was 1.67 (95% CI, 1.49-1.87).

**Conclusions and Relevance:**

Concordant receipt of ANS and postnatal life support was associated with significantly higher survival and survival without major morbidities at 22 through 25 weeks’ gestation compared with life support alone. Although statistically higher with ANS, survival without major morbidities remains low at 22 and 23 weeks. There is an opportunity to reevaluate national obstetric guidelines, allowing for shared decision making at the edge of viability with concordant obstetrical and neonatal treatment plans.

## Key Points

**Question:**

For infants born at the edge of viability who received postnatal life support, was the administration of antenatal steroids associated with higher rates of survival?

**Finding:**

In a cohort study of 33 472 infants born at 22 to 25 weeks’ gestation between 2012 and 2016, the concordant receipt of antenatal steroids and postnatal life support was significantly associated with higher rates of survival compared with postnatal life support alone.

**Meaning:**

There is an opportunity for reevaluation of national guidelines, allowing for shared decision making with concordant obstetrical and neonatal treatment plans.

## Teaser

This cohort study of US infants receiving postnatal life support at 22 to 25 weeks’ gestation examines whether exposure to antenatal steroids is associated with survival to hospital discharge and survival without major morbidities.

## Introduction

Decisions surrounding the perinatal goals of care for infants born extremely preterm (<28 weeks) are highly controversial. With advancing neonatal intensive care technology and capabilities, an increasing number of infants born at 22 to 25 weeks’ gestation receive postnatal life support. This increase reflects a shifting limit of viability with a focus on shared decision making with families.^1,2,3,4^ We currently do not have consensus in the United States on viability, and importantly additional factors other than gestational age are used for individual counseling.^5^ There is significant hospital-level variation in the provision of postnatal life support at 22 to 24 weeks’ gestation.^6^ With careful consideration of the risk of death and morbidities including severe neurodevelopmental impairment, some institutional guidelines allow informed parents to choose palliative comfort care for their extremely preterm infants up through 25 6/7 weeks’ gestation.^4^

In an effort to provide health care professionals with a unified framework both for medical decision making and counseling families, the Eunice Kennedy Shriver National Institute of Child Health and Human Development, the Society for Maternal-Fetal Medicine (SMFM), the American Academy of Pediatrics, and the American College of Obstetricians and Gynecologists (ACOG) convened a joint workshop in 2014 to review the best available evidence for obstetrical and neonatal interventions in the periviable period.^7^ The general guidance was to consider the use of antenatal steroids (ANS) starting at 22 0/7 weeks if delivery at or later than 23 weeks was anticipated, and not to recommend postnatal life support until 23 0/7 weeks unless a fetus was considered potentially viable based on individual circumstances.

Incorporating new data on neonatal survival and morbidities, ACOG and SMFM released updated guidance with an important shift in the concordance of care.^8^ They recommended deferring ANS until 23 0/7 weeks’ gestation, while allowing for neonatal assessment for resuscitation starting at 22 0/7 weeks’ gestation, based on a family’s decision. Although these guidelines try to incorporate families, they recommend discordant care at the edge of viability. Families may be offered and choose postnatal life support at 22 weeks’ gestation, but the obstetric community does not currently recommend giving ANS in preparation of preterm birth and resuscitation at this gestation.

The most recent Cochrane systematic reviews assessing the efficacy of ANS in extreme prematurity,^9,10^ found limited relevant data to address this question. The landmark study by Liggins and Howie^11^ published in 1972 is the only randomized clinical trial with fetuses less than 26 weeks’ gestation at the first dose of ANS (n = 27). We currently do not have trial-level data to adequately test the benefit of ANS at gestations less than 26 weeks.^10^

The current ACOG and SMFM guidelines to consider administration of ANS at 23 weeks but not at 22 weeks are not based on evidence from randomized clinical trials, but rather consensus and observational studies with limited statistical power.^7,12,13,14,15,16,17,18,19,20,21,22^ Medical professionals and families struggle with the quandary of limited relevant data on survival and morbidities,^23^ and the recommended discordant care at 22 weeks.

Vermont Oxford Network (VON) is uniquely suited to address this question in an extensive cohort largely representative of national practice with outcomes reflecting current pragmatic care. Our objective was to estimate the proportion of infants receiving postnatal life support at 22 to 25 weeks’ gestation who had exposure to ANS, and to examine if the provision of ANS was associated with a higher rate of survival to hospital discharge and survival without major morbidities.

## Methods

Vermont Oxford Network is a voluntary worldwide collaborative of hospitals working to improve the quality and safety of medical care for newborn infants and their families through a coordinated program of research, education, and quality improvement projects. We studied 33 472 infants born between January 1, 2012, and December 31, 2016, at 431 US VON member hospitals with level III and IV neonatal intensive care units (NICUs) that perform surgery on neonates (eTable 2 in the Supplement). This analysis includes standardized data collected for liveborn infants born at 22 0/7 weeks’ to 25 6/7 weeks’ gestation, including those who died in the delivery room, and without a minimum birth weight.

We excluded 1329 infants with recognized syndromes or major congenital malformations, 121 infants with missing data, and 55 infants with implausible birth weights, defined as greater than 4 SD above the mean by week and sex.

### Data Collection

Local staff collected infant data using uniform definitions^24^ until death, discharge home, or transfer to other hospitals. Race and ethnicity were abstracted from interviews with the mother, or review of the birth certificate or medical record, in that order of preference. All data underwent automated checks for quality and completeness at the time of submission. The University of Vermont Committee on Human Research determined that the use of the VON Research Repository for this analysis was not human subjects research, and waiver of informed consent was granted. Our reporting of this study followed the Strengthening the Reporting of Observational Studies in Epidemiology (STROBE) reporting guideline.^25,26^

### Definitions

Gestational age at birth was determined by the best estimate using the following hierarchy: obstetrical measures based on last menstrual period, obstetrical parameters, and prenatal ultrasonography followed by neonatologist’s estimate based on physical criteria and examination.^24^ Small for gestational age status was defined within categories of sex, race, ethnicity, and multiple birth as birth weight below the 10th percentile on the basis of smoothed curves constructed using the US Natality Data set.^27^ Apgar scores were assigned by the clinical team as a standard assessment for infants after birth. An Apgar score of 3 or less was categorized as low, describing an infant in poor condition.^28,29,30,31^

Infants were considered to have exposure to ANS if betamethasone, dexamethasone, or hydrocortisone was administered intramuscularly or intravenously to the mother during pregnancy at any time prior to delivery.^24^

Infants were considered to have received postnatal life support if they received any of the following interventions: respiratory support (including face mask ventilation, nasal continuous positive airway pressure, endotracheal intubation, surfactant therapy, or mechanical ventilation), chest compressions, or epinephrine.

### Outcomes

The primary outcome was survival to hospital discharge. Infants transferred between hospitals were tracked for survival status until discharge.

Secondary outcomes included major morbidities among survivors: chronic lung disease (CLD); severe intraventricular hemorrhage; cystic periventricular leukomalacia; necrotizing enterocolitis; culture-confirmed infection; severe retinopathy of prematurity; and the composite outcome of survival to discharge without major morbidities. Chronic lung disease was defined as oxygen use at 36 weeks’ postmenstrual age (PMA) or at discharge for infants 34 to 35 weeks’ PMA.^32^ Oxygen use was determined by the infant’s clinical team, and does not necessarily imply that a physiological test for oxygen requirement was completed at 36 weeks’ PMA.^33,34^ Severe intraventricular hemorrhage was defined as grades 3 and 4.^35^ Severe retinopathy of prematurity was defined as stages 3 to 5.^36^ Culture-confirmed infection was defined as bacterial or fungal sepsis and/or meningitis at any time during the NICU admission based on positive blood or cerebrospinal fluid cultures.^24^ Necrotizing enterocolitis was diagnosed by the clinical team at surgery, postmortem examination, or clinically and radiographically using standard criteria from the VON Manual of Operations definitions.^24^

### Statistical Analysis

We calculated overall rates of postnatal life support, with and without exposure to ANS, by gestational age at birth. We calculated risk ratios (RRs) for survival and each secondary outcome, to compare infants with and without exposure to ANS among those who received postnatal life support. To estimate adjusted RRs (aRRs) for survival and survival without major morbidities, we used log binomial models with generalized estimating equations, adjusting for prenatal care, maternal hypertension, chorioamnionitis, maternal race and ethnicity, multiple births, sex, small for gestational age status, mode of delivery, and hospital-level clustering.^37^ Prespecified subgroups included gestational age groups by week. The RRs for all covariates included in the models are provided in eTable 1 in the Supplement. The R software package, version 3.3.2 (The R Foundation) was used for all analyses.^38,39,40^

### Sensitivity Analyses

We completed a sensitivity analysis based on the E value^41^ to assess the minimum strength of association, on the RR scale, that an unmeasured confounder would need to have with both the treatment and outcome to explain away the association of ANS and survival, and ANS and survival without major morbidities in our model. In addition, we considered the possibility that annual hospital volume of very low birth weight deliveries and NICU level may explain some of the association between ANS and survival; this possibility has been proposed previously.^42,43^

## Results

### Mother and Infant Characteristics

**Figure 1.**
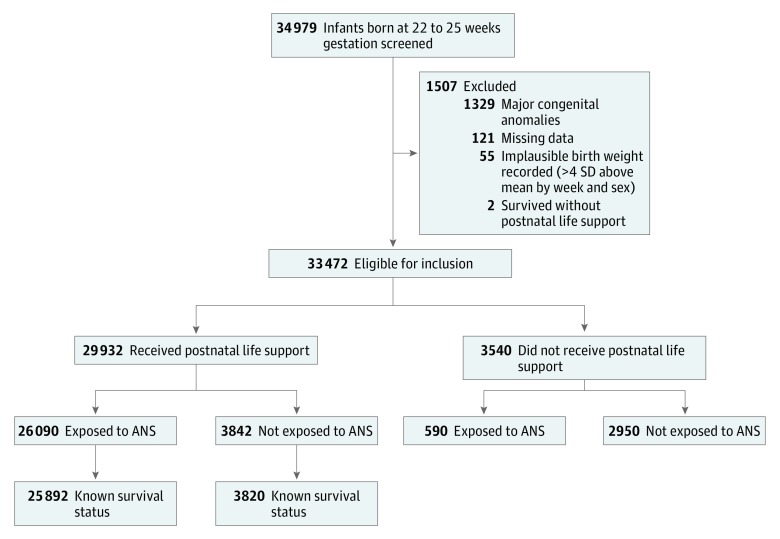
Flow Diagram for Cohort Flow diagram of population screened, inclusion in cohort, receipt of postnatal life support, exposure to antenatal steroids, and known survival status. ANS indicates antenatal steroids; SD, standard deviation.

A total of 33 472 infants were eligible for inclusion (infants per hospital: median, 66; range, 1-366). Of the 3540 infants who did not receive postnatal life support, 590 had ANS exposure. Lack of postnatal life support resulted in death in all of these 3540 infants. Of the remaining 29 932 infants, 26 090 (87.2%) received postnatal life support with ANS exposure and 3842 (12.8%) received postnatal life support without ANS exposure (Figure 1). The infants who received postnatal life support were 51.9% male, with mean (SD) gestational age of 24.12 (0.86) weeks and mean birth weight of 668 (140) g.

Mothers treated with ANS were more likely to identify with the white racial group, to have received prenatal care, and to have hypertension at all weeks of gestation. Mothers identifying as non-Hispanic and diagnosed with chorioamnionitis were more likely to receive ANS at 23, 24, and 25 weeks. Infants with ANS exposure were more likely to have had cesarean delivery and to be small for gestational age at birth, and less likely to be singleton births or have a 1-minute Apgar score of 3 or less (Table 1).

**Table 1.** Mother and Infant Characteristics

Variable	No. (%)									
	22 wk		23 wk		24 wk		25 wk		22-25 wk	
	No ANS (n = 504)	ANS (n = 554)	No ANS (n = 1104)	ANS (n = 5267)	No ANS (n = 1127)	ANS (n = 9381)	No ANS (n = 1107)	ANS (n = 10 888)	No ANS (n = 3842)	ANS (n = 26 090)
Mother										
Received prenatal care	460 (91.3)	524 (94.6)	987 (89.4)	5100 (96.8)	977 (86.7)	9113 (97.1)	969 (87.5)	10 613 (97.5)	3393 (88.3)	25 350 (97.2)
Race										
Black	232 (46.5)	233 (42.4)	471 (43.1)	2034 (38.9)	445 (39.7)	3320 (35.6)	450 (40.9)	3618 (33.4)	1598 (41.9)	9205 (35.5)
White	210 (42.1)	247 (44.9)	492 (45.0)	2462 (47.1)	514 (45.8)	4617 (49.6)	490 (44.5)	5645 (52.2)	1706 (44.7)	12 971 (50.1)
Other	57 (11.4)	70 (12.7)	130 (11.9)	732 (14.0)	163 (14.5)	1378 (14.8)	161 (14.6)	1559 (14.4)	511 (13.4)	3739 (14.4)
Hispanic	90 (17.9)	110 (20.0)	222 (20.3)	999 (19.0)	236 (21.1)	1802 (19.3)	239 (21.6)	2044 (18.8)	787 (20.6)	4955 (19.1)
Hypertension	38 (7.6)	54 (9.8)	81 (7.4)	736 (14.0)	167 (15.0)	1857 (19.8)	175 (15.8)	2715 (25.0)	461 (12.1)	5362 (20.6)
Chorioamnionitis	151 (30.1)	149 (27.0)	246 (22.6)	1567 (29.9)	173 (15.5)	2469 (26.4)	174 (15.8)	2443 (22.5)	744 (19.5)	6628 (25.5)
Infant										
Birth weight, mean (SD), g	515 (74.0)	579 (90.1)	673 (110.7)	776 (139.5)	510 (80.7)	577 (90.0)	650 (114.7)	741 (140.4)	655 (144.2)	670 (138.7)
Singleton birth	381 (75.6)	397 (71.7)	864 (78.3)	3782 (71.8)	900 (79.9)	7177 (76.5)	918 (82.9)	8312 (76.3)	3063 (79.7)	19 668 (75.4)
Male	265 (52.6)	309 (55.8)	573 (51.9)	2661 (50.5)	597 (53.0)	4883 (52.1)	615 (55.6)	5624 (51.7)	2050 (53.4)	13 477 (51.7)
Small for gestational age	5 (1.0)	10 (1.8)	24 (2.2)	129 (2.5)	33 (2.9)	459 (4.9)	48 (4.3)	759 (7.0)	110 (2.9)	1357 (5.2)
Apgar score ≤3 at 1 min^a^	405 (80.5)	411 (74.2)	839 (76.0)	3298 (62.6)	718 (63.8)	4834 (51.5)	650 (58.7)	4595 (42.2)	2612 (68.0)	13 138 (50.4)
Cesarean delivery	82 (16.3)	175 (31.6)	424 (38.4)	2842 (54.0)	747 (66.3)	6561 (69.9)	758 (68.5)	7916 (72.7)	2011 (52.3)	17 493 (67.0)

Abbreviation: ANS, antenatal steroids.

^a^ Apgar scores describe a standardized assessment for infants after birth, with scores reported at 1 minute and 5 minutes after birth, with a possible score of 0 to 10. There are 5 components (scored 0, 1, or 2 at each time): heart rate, respiratory effort, reflex irritability, and muscle tone and color. An Apgar score of 3 or less is categorized as low, describing an infant in poor condition.^28,29,30,31^

**Figure 2.**
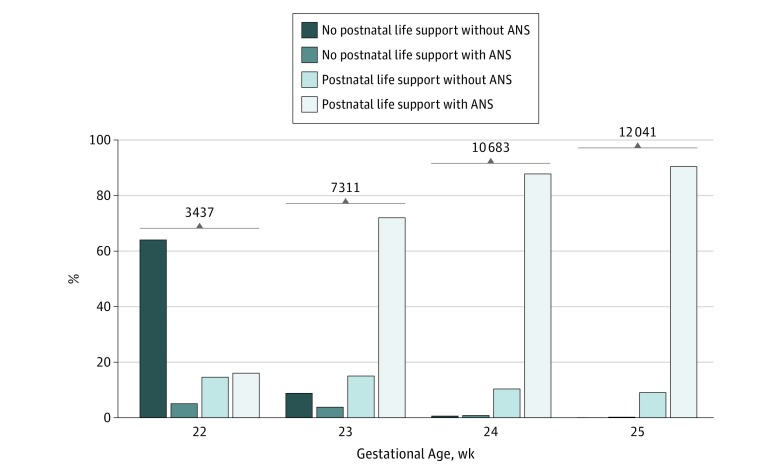
Proportion of Infants Receiving Postnatal Life Support, by Gestational Age at Birth The mean percentage of infants receiving no postnatal life support without antenatal steroids (ANS) exposure, no postnatal life support with ANS exposure, postnatal life support without ANS exposure, and postnatal life support with ANS exposure are shown for each gestational age, 22 to 25 weeks. Numbers above bars indicate sample size at each gestational age.

The proportion of eligible infants who received postnatal life support varied by gestational age week at birth: 30.8% at 22 weeks, 87.1% at 23 weeks, 98.4% at 24 weeks, and 99.6% at 25 weeks (Figure 2). Of the infants receiving postnatal life support, the proportion exposed to ANS increased with advancing gestational age: 52.4% at 22 weeks, 82.7% at 23 weeks, 89.3% at 24 weeks, and 90.8% at 25 weeks.

### Primary Outcome

Overall, 1981 of 3820 infants (51.9%) who received postnatal life support without ANS exposure survived to hospital discharge, compared with 18 717 of 25 892 infants (72.3%) who received postnatal life support with ANS exposure (aRR, 1.37; 95% CI, 1.32-1.42). At each week of gestation, infants who received both ANS and postnatal life support were more likely to survive than infants who received postnatal life support alone: 38.5% vs 17.7% at 22 weeks (aRR, 2.11; 95% CI, 1.68-2.65), 55.4% vs 35.6% at 23 weeks (aRR, 1.54; 95% CI, 1.40-1.70), 71.3% vs 59.6% at 24 weeks (aRR, 1.18; 95% CI, 1.12-1.25), and 83.0% vs 75.7% at 25 weeks (aRR, 1.11; 95% CI, 1.07-1.14) (Table 2).

**Table 2.** Survival Rates, by Gestational Age at Birth

Gestational Age, wk	No. of Survivors/Total No. of Infants (%)		RR (95% CI)	aRR (95% CI)^a^
	Postnatal Life Support Alone	Postnatal Life Support With ANS Exposure		
22	89/503 (17.7)	210/546 (38.5)	2.17 (1.75-2.70)	2.11 (1.68-2.65)
23	391/1097 (35.6)	2884/5210 (55.4)	1.55 (1.43-1.69)	1.54 (1.40-1.70)
24	667/1119 (59.6)	6640/9312 (71.3)	1.20 (1.14-1.26)	1.18 (1.12-1.25)
25	834/1101 (75.7)	8983/10 825 (83.0)	1.10 (1.06-1.13)	1.11 (1.07-1.14)
22-25	1981/3820 (51.9)	18 717/25 892 (72.3)	1.39 (1.35-1.44)	1.37 (1.32-1.42)

Abbreviations: ANS, antenatal steroids; aRR, adjusted risk ratio; RR, risk ratio.

^a^ Adjusted for prenatal care, maternal hypertension, chorioamnionitis, maternal race and ethnicity, multiple births, sex, small for gestational age status, mode of delivery, and hospital-level clustering.

### Secondary Outcomes

For infants at 22 to 25 weeks’ gestation receiving postnatal life support, exposure to ANS was associated with improved survival without major morbidities (3777 of 25 833 [14.6%] with ANS exposure vs 347 of 3806 [9.1%] without ANS exposure; aRR, 1.67; 95% CI, 1.49-1.87). This finding was consistent at each week of gestation. Although the largest associated benefit for ANS exposure was at the lowest gestational age, the rates of survival without major morbidities remained low: 4.4% vs 1.0% at 22 weeks, 5.9% vs 2.8% at 23 weeks, 11.4% vs 9.5% at 24 weeks, and 22.2% vs 18.8% at 25 weeks (Table 3). The most common major morbidity at each gestational age, and overall, was CLD, which affected approximately 65% of surviving infants, and was unchanged with the receipt of ANS. Among survivors, receipt of ANS was associated with an improved survival without severe intraventricular hemorrhage (RR, 1.16; 95% CI, 1.13-1.19) and survival without periventricular leukomalacia (RR, 1.03; 95% CI, 1.02-1.05). All secondary outcomes by gestational age at birth are shown in Table 3.

**Table 3.** Secondary Outcomes, by Gestational Age at Birth

Outcome	No. of Cases/No. of Infants (%)		RR (95% CI)	aRR (95% CI)^a^
	Postnatal Life Support Alone	Postnatal Life Support With ANS Exposure		
22 wk				
Survival without major morbidities	5/502 (1.0)	24/551 (4.4)	4.37 (1.68-11.37)	4.35 (1.84-10.28)^b^
Survival without chronic lung disease^c^	16/82 (19.5)	53/197 (26.9)	1.38 (0.84-2.26)	
Survival without severe intraventricular hemorrhage^d^	58/85 (68.2)	160/208 (76.9)	1.13 (0.96-1.33)	
Survival without cystic periventricular leukomalacia^e^	79/87 (90.8)	190/208 (91.3)	1.01 (0.93-1.09)	
Survival without necrotizing enterocolitis^f^	77/89 (86.5)	188/209 (90.0)	1.04 (0.95-1.14)	
Survival without severe retinopathy of prematurity^g^	49/83 (59.0)	127/200 (63.5)	1.08 (0.87-1.32)	
Survival without culture-confirmed infection^f^	46/89 (51.7)	124/210 (59.0)	1.14 (0.91-1.44)	
23 wk				
Survival without major morbidities	31/1099 (2.8)	307/5239 (5.9)	2.08 (1.44-2.99)	2.19 (1.48-3.25)
Survival without chronic lung disease^c^	90/355 (25.4)	663/2695 (24.6)	0.97 (0.80-1.17)	
Survival without severe intraventricular hemorrhage^d^	249/381 (65.4)	2260/2847 (79.4)	1.21 (1.13-1.31)	
Survival without cystic periventricular leukomalacia^e^	351/389 (90.2)	2691/2864 (94.0)	1.04 (1.01-1.08)	
Survival without necrotizing enterocolitis^f^	343/391 (87.7)	2607/2884 (90.4)	1.03 (0.99-1.07)	
Survival without severe retinopathy of prematurity^g^	216/363 (59.5)	1672/2696 (62.0)	1.04 (0.95-1.14)	
Survival without culture-confirmed infection^f^	245/389 (63.0)	1861/2880 (64.6)	1.03 (0.95-1.11)	
24 wk				
Survival without major morbidities	106/1115 (9.5)	1060/9299 (11.4)	1.20 (0.99-1.45)	1.27 (1.04-1.56)
Survival without chronic lung disease^c^	206/604 (34.1)	1905/6248 (30.5)	0.89 (0.80-1.00)	
Survival without severe intraventricular hemorrhage^d^	479/656 (73.0)	5611/6560 (85.5)	1.17 (1.12-1.23)	
Survival without cystic periventricular leukomalacia^e^	600/657 (91.3)	6268/6595 (95.0)	1.04 (1.02-1.07)	
Survival without necrotizing enterocolitis^f^	610/666 (91.6)	6104/6634 (92.0)	1.00 (0.98-1.03)	
Survival without severe retinopathy of prematurity^g^	455/613 (74.2)	4451/6259 (71.1)	0.96 (0.91-1.01)	
Survival without culture-confirmed infection^f^	453/665 (68.1)	4693/6629 (70.8)	1.04 (0.98-1.10)	
25 wk				
Survival without major morbidities	205/1090 (18.8)	2386/10 744 (22.2)	1.18 (1.04-1.34)	1.26 (1.10-1.44)
Survival without chronic lung disease^c^	345/783 (44.1)	3614/8607 (42.0)	0.95 (0.88-1.04)	
Survival without severe intraventricular hemorrhage^d^	660/823 (80.2)	7918/8857 (89.4)	1.11 (1.08-1.15)	
Survival without cystic periventricular leukomalacia^e^	779/826 (94.3)	8581/8932 (96.1)	1.02 (1.00-1.04)	
Survival without necrotizing enterocolitis^f^	787/834 (94.4)	8346/8981 (92.9)	0.98 (0.97-1.00)	
Survival without severe retinopathy of prematurity^g^	644/772 (83.4)	7052/8540 (82.6)	0.99 (0.96-1.02)	
Survival without culture-confirmed infection^f^	638/833 (76.6)	6978/8962 (77.9)	1.02 (0.98-1.06)	
22-25 wk				
Survival without major morbidities	347/3806 (9.1)	3777/25 833 (14.6)	1.60 (1.44-1.78)	1.67 (1.49-1.87)
Survival without chronic lung disease^c^	657/1824 (36.0)	6235/17 747 (35.1)	0.98 (0.91-1.04)	
Survival without severe intraventricular hemorrhage^d^	1446/1945 (74.3)	15 949/18 472 (86.3)	1.16 (1.13-1.19)	
Survival without cystic periventricular leukomalacia^e^	1809/1959 (92.3)	17 730/18 599 (95.3)	1.03 (1.02-1.05)	
Survival without necrotizing enterocolitis^f^	1817/1980 (91.8)	17 245/18 708 (92.2)	1.00 (0.99-1.02)	
Survival without severe retinopathy of prematurity^g^	1364/1831 (74.5)	13 302/17 695 (75.2)	1.01 (0.98-1.04)	
Survival without culture-confirmed infection^f^	1382/1976 (69.9)	13 656/18 681 (73.1)	1.05 (1.01-1.08)	

Abbreviations: ANS, antenatal steroids; aRR, adjusted risk ratio; RR, risk ratio.

^a^ Adjusted for prenatal care, maternal hypertension, chorioamnionitis, maternal race and ethnicity, multiple births, sex, small for gestational age status, mode of delivery, and hospital-level clustering.

^b^ Risk adjustment model for this outcome at 22 weeks does not include small for gestational age status or Hispanic ethnicity due to sample size limitation.

^c^ Among surviving infants discharged or transferred at 34 weeks or later.

^d^ Among surviving infants who received cranial imaging within 28 days of birth.

^e^ Among surviving infants who received cranial imaging before discharge.

^f^ Among surviving infants.

^g^ Among surviving infants who received an eye examination before discharge.

### Sensitivity Analyses

The E value for our analysis of survival overall was 2.09 (lower confidence limit, 1.97), and for survival without major morbidities was 2.73 (lower confidence limit, 2.35). The observed aRR of 1.37 for survival and 1.67 for survival without major morbidities for infants born at 22 through 25 weeks’ gestation exposed to ANS could be explained away by an unmeasured confounder that was associated with both the treatment and the outcomes each by an RR of 2.09 for survival and an RR of 2.73 for survival without major morbidities, above and beyond the measured confounders in our analysis, but weaker confounding could not do so. Furthermore, there was no significant relationship between annual hospital volume of very low-birth-weight deliveries or NICU level and survival in our model.

## Discussion

In this large US-based prospective cohort study involving infants born at 22 to 25 weeks’ gestation, the combination of postnatal life support with ANS exposure was associated with a significantly higher incidence of survival and survival without major morbidities, overall and at each gestational age 22 through 25 weeks, than postnatal life support alone. Overall, 69.7% of the infants at 22 to 25 weeks’ gestation receiving postnatal life support survived to hospital discharge (51.9% without ANS exposure; 72.3% with ANS exposure), yet few survived without a major morbidity (9.1% without ANS exposure; 14.6% with ANS exposure). This study, which included more than 1000 infants born at 22 weeks’ gestational age who received postnatal life support, to our knowledge, is the largest published cohort of this population to date, affording the statistical power to examine the association at the current edge of viability, an age in which relevant data are lacking. Although the survival of 22-week infants in our cohort was twice as high with ANS exposure, 38.5% vs 17.7% without ANS exposure, the rate of survival without major morbidities remained very low, 4.4% with ANS exposure and 1.0% without ANS exposure.

As a pragmatic analysis, ANS exposure was defined as maternal receipt of ANS at any time prior to delivery. Previous multicenter network analyses have chosen any ANS as often the prescribed course is not completed, and because any ANS represents a more conservative estimate of the effects of ANS treatment than a complete course.^14^ The evidence shows a dose-dependent protective effect for ANS including protection against death or neurodevelopmental impairment in extremely preterm infants,^44,45,46^ with a rapid decline in mortality seen at ANS to birth intervals of less than 12 hours.^47^ This data supports our approach, and the pragmatic recommendation not to forgo treatment with ANS solely based on the assumption that a full course of therapy will not be completed.

Decisions regarding inclusion of major morbidities were informed by evidence of increased risk of a late death or neurosensory impairment in extremely low-birth-weight infants who survived to 36 weeks’ PMA.^48,49^ For this reason, CLD, severe intraventricular hemorrhage, cystic periventricular leukomalacia, severe retinopathy of prematurity, necrotizing enterocolitis, and culture-confirmed infection were included, recognizing that each diagnosis carries a different risk for subsequent development of neurodevelopmental impairment, and may be valued differently by medical professionals and families.^50,51^ This cohort was followed to hospital discharge, lacking important long-term follow-up.

The improved survival in our 2012 to 2016 cohort compared with previously published work^6,12,17,52,53,54^ likely reflects continued improved survival outcomes over time. We also show higher rates of extremely preterm infants receiving postnatal life support (30.8% at 22 weeks; 87.1% at 23 weeks) compared with the 2006 to 2011 Eunice Kennedy Shriver National Institute of Child Health and Human Development cohort (22.1% at 22 weeks; 71.8% at 23 weeks) by Rysavy et al,^6^ potentially owing to continued trends over time, or a larger cohort more representative of national practice. Our finding of the differential exposure to ANS at 22 to 25 weeks among racial and ethnic groups confirms previous findings by Carlo et al,^17^ and remains a necessary area for future research and advocacy. Although ANS treatment has not been found to increase the risk of chorioamnionitis,^10^ the differential exposure in our cohort at 23 to 25 weeks likely reflects confounding by indication as often this diagnosis is made in the context of hospital admission, obstetrical care, and counseling. Although mothers treated with ANS had higher rates of chorioamnionitis, we do not know the time relationship between infection diagnosis and receipt of ANS, and possible association with premature prolonged rupture of membranes. Importantly, at each gestational age week and overall, survival without culture-confirmed infection was greater for infants with ANS exposure, compared with postnatal life support alone.

### Limitations

A major limitation of examining the association between ANS exposure and survival outcomes with an observational cohort study is confounding by indication. Although statistical methods allow adjustment for confounding, there are unmeasured differences between the maternal-fetal dyads who receive ANS and those who do not. We lack data on the time from maternal admission to delivery and indication for cesarean delivery, which would allow for a better estimate of precipitous and emergency deliveries. There could potentially be more high-risk pregnancies and deliveries in the group lacking ANS exposure, causing unmeasured elevated baseline mortality risk in this group. There also may be a nuanced approach with selection bias of active perinatal management, including the receipt of ANS, based on the perceived prognosis of the fetus based on factors such as estimated fetal weight and sex.^55^ Residual confounding likely persists, but sensitivity analyses suggest that this is unlikely to explain the study findings.

Considering differential neonatal treatment, previous studies have shown that periviable infants with ANS exposure are more likely to receive aggressive treatment in the delivery room, to survive the delivery room, and to survive to hospital discharge.^56,57^ Importantly, however, as chest compressions and epinephrine have been noted previously as prognostic markers for adverse neurodevelopmental outcomes, families may decide in counseling before birth to decline trials of these interventions if initial ventilatory support fails to stabilize the heart rate of their periviable infant in a normal range.^58,59,60^ We therefore used a composite measure to define postnatal life support adapted from previously published work by Rysavy et al.^6^ The study lacks data on the proportion of patients that could have received additional care if aggressive or full resuscitation was requested by all families. While the study focused on the care that the mother and infant received, the underlying intentions and understanding of the family members are unknown. As the analysis focused on liveborn infants who received postnatal life support, we do not know the quantity of pregnancies that had outcomes of termination, intrauterine fetal demise, or stillbirth, and any association with ANS receipt or nonintervention.^2,61^

Finally, the infants included in this analysis are inborn at US VON member hospitals with level III and IV NICUs, consistent with the American Academy of Pediatrics Committee on the Fetus and Newborn’s policy statement on levels of neonatal care and recommendations for births at risk-appropriate sites.^62,63^ We caution that the findings from this analysis may not be generalizable to outborn infants, and infants born in settings with different intensive care services. Although the hospitals included in this analysis varied in volume of extremely preterm infants, volume did not have a significant relationship with survival in our model. Elsewhere the outcomes of infants treated in US NICUs have been shown to vary by the number of very low-birth-weight infants admitted.^43,64^ Our inclusive approach may yield a more conservative estimate of the overall survival benefit of ANS, but may underestimate outcomes achieved in some high-volume NICUs. Vermont Oxford Network is a voluntary collaboration of hospitals. Although the VON database includes nearly 90% of the very low-birth-weight infants born in the United States and is a cohort largely representative of national practice, it is not necessarily a nationally representative sample such as seen in countries with a national registry.

These limitations, however, must be considered in the context of appropriate and feasible clinical research. Current guidelines endorsed by SMFM and ACOG recommend ANS for anticipated preterm birth between 24 and 33 6/7 weeks’ gestation with consideration starting at 23 weeks, based on a family’s decision regarding resuscitation. Although ideally a randomized clinical trial would be performed at 22 to 25 weeks’ gestation to fill the data gap currently answered by observational studies and expert opinion, it is unlikely. Antenatal steroids have been recommended with limited evidence at gestational ages of 24 to 25 weeks since the early 1990s and have become standard of care.^8^ A large, recent, high-quality, prospective cohort with outcomes largely reflecting national pragmatic care practices is likely the highest level of evidence currently possible to ethically address this challenging question.

Interventions at the edge of viability raise difficult considerations about the best interests of the infant and family, and about the just distribution of limited health care resources.^4,65^ We are not advocating for a specific approach to interventions at the edge of viability or a change in when postnatal life support is offered in counseling and shared decision making with families. Rather, we are pointing out that there is currently discordance in the recommended obstetric and neonatal interventions at the edge of viability, which is likely leading to a discrepancy between current practice and existing guidelines. It is unclear how discordant recommendations are understood by clinicians, incorporated into institutional guidelines, and then presented to parents and family members in discussions on goals of care. Regardless of the guidelines at the edge of viability, these decisions will remain extremely difficult and must be based on shared decision making between health care professionals and the families we serve.^66^

## Conclusions

We found that concordant receipt of ANS and postnatal life support was associated with significantly higher survival and survival without major morbidities at 22 through 25 weeks’ gestation compared with life support alone. Although statistically higher with ANS, survival without major morbidities remains low at 22 and 23 weeks. If informed families are offered and choosing postnatal life support with the goal of survival and survival without significant neonatal morbidities, then ANS should also be part of that decision.

## Notes

Supplement. **eTable 1.** Adjusted Risk Ratios for Model Covariates **eTable 2.** List of Contributing VON Member Hospitals
