# Supplementary material for: Association of Antenatal Steroid Exposure With Survival Among Infants Receiving Postnatal Life Support at 22 to 25 Weeks’ Gestation
Source: JAMA Netw Open. 2018 Oct 12;1(6):e183235. doi: 10.1001/jamanetworkopen.2018.3235 (PMC6324435; doi:10.1001/jamanetworkopen.2018.3235)
Supplement: Supplement. — eTable 1. Adjusted Risk Ratios for Model Covariates eTable 2. List of Contributing VON Member Hospitals [file jamanetwopen-1-e183235-s001.pdf]

Supplementary Online Content

Ehret DEY, Edwards EM, Greenberg LT, et al. Association of antenatal steroid exposure with survival among infants receiving postnatal life support at 22 to 25 weeks' gestation. *JAMA Netw Open*. 2018;1(6):e183235. doi:10.1001/jamanetworkopen.2018.3235

eTable 1. Adjusted Risk Ratios for Model Covariates

eTable 2. List of Contributing VON Member Hospitals

This supplementary material has been provided by the authors to give readers additional information about their work.

© 2018 Ehret DEY et al. *JAMA Networ Open*.

eTable 1. Adjusted Risk Ratios for Model Covariates

	**Prenatal care**	**Maternal Hypertension**	**Chorioamninitis**	**Maternal race: black**	**Maternal race: other**	**Hispanic**	**Multiple birth**	**Male sex**	**SGA**	**Vaginal delivery**
**Survival**										
22 Weeks	1.05 (0.74-1.49)	0.86 (0.64-1.17)	0.98 (0.79-1.22)	1.28 (1.01-1.62)	0.80 (0.58-1.11)	1.50 (1.16-1.93)	0.70 (0.53-0.93)	0.76 (0.64-0.90)	0.37 (0.13-1.08)	0.82 (0.68-1.00)
23 Weeks	1.03 (0.92-1.15)	1.00 (0.93-1.08)	1.05 (1.00-1.11)	1.10 (1.04-1.17)	0.95 (0.87-1.05)	1.13 (1.06-1.21)	0.86 (0.81-0.92)	0.90 (0.86-0.95)	0.36 (0.25-0.53)	0.95 (0.90-1.00)
24 Weeks	1.06 (0.99-1.13)	0.94 (0.91-0.97)	1.04 (1.01-1.07)	1.05 (1.02-1.08)	1.00 (0.96-1.04)	1.03 (1.00-1.07)	0.93 (0.90-0.96)	0.94 (0.92-0.96)	0.55 (0.49-0.62)	1.00 (0.97-1.03)
25 Weeks	1.03 (0.98-1.08)	0.98 (0.96-1.00)	0.99 (0.97-1.01)	1.03 (1.01-1.05)	1.01 (0.98-1.04)	1.03 (1.00-1.05)	0.98 (0.96-1.01)	0.96 (0.95-0.98)	0.65 (0.60-0.70)	1.02 (1.00-1.04)
22-25 Weeks	1.04 (1.00-1.08)	0.99 (0.97-1.01)	1.01 (0.99-1.03)	1.03 (1.01-1.05)	1.00 (0.97-1.03)	1.04 (1.02-1.07)	0.92 (0.90-0.94)	0.94 (0.93-0.96)	0.61 (0.57-0.65)	0.93 (0.92-0.95)
**Survival without Morbidities**										
22 Weeks	0.64 (0.24-1.68)	1.16 (0.48-2.84)	0.63 (0.29-1.40)	2.55 (1.12-5.80)	1.50 (0.51-4.38)	1.89 (0.75-4.78)	0.31 (0.11-0.89)	0.86 (0.40-1.86)		0.67 (0.36-1.27)
23 Weeks	0.76 (0.46-1.25)	1.05 (0.76-1.45)	0.91 (0.69-1.18)	1.39 (1.04-1.86)	0.98 (0.69-1.39)	1.84 (1.34-2.52)	0.65 (0.47-0.88)	0.79 (0.64-0.98)	0.23 (0.04-1.27)	0.85 (0.69-1.05)

© 2018 Ehret DEY et al. *JAMA Networ Open*.

24 Weeks	0.62 (0.49-0.79)	0.86 (0.73-1.01)	1.14 (0.99-1.31)	1.39 (1.21-1.60)	1.08 (0.88-1.33)	1.24 (1.02-1.51)	0.91 (0.79-1.04)	0.74 (0.67-0.81)	0.32 (0.20-0.50)	1.03 (0.91-1.16)
25 Weeks	0.89 (0.74-1.08)	0.73 (0.66-0.80)	1.03 (0.93-1.13)	1.29 (1.19-1.41)	1.01 (0.90-1.14)	1.10 (1.00-1.22)	0.83 (0.75-0.92)	0.77 (0.72-0.83)	0.31 (0.23-0.43)	1.10 (1.02-1.19)
22-25 Weeks	0.79 (0.68-0.91)	0.83 (0.76-0.91)	1.01 (0.92-1.10)	1.26 (1.16-1.37)	1.04 (0.93-1.16)	1.17 (1.07-1.29)	0.78 (0.71-0.85)	0.77 (0.73-0.81)	0.33 (0.25-0.42)	0.90 (0.84-0.96)

© 2018 Ehret DEY et al. *JAMA Networ Open*.

eTable 2. List of Contributing VON Member Hospitals

Hospital Name	City	State
University of Alabama at Birmingham	Birmingham	Alabama
Huntsville Hospital	Huntsville	Alabama
USA Children's and Women's Hospital	Mobile	Alabama
Children's Hospital at Providence, Alaska, The	Anchorage	Alaska
Banner Thunderbird Medical Center	Glendale	Arizona
Cardon Children's Medical Center	Mesa	Arizona
Arizona Children's Cent Maricopa Integrated Hlth.	Phoenix	Arizona
Banner University Medical Center Phoenix	Phoenix	Arizona
Phoenix Children's Hospital	Phoenix	Arizona
St. Joseph's Hospital and Medical Center	Phoenix	Arizona
HonorHealth Scottsdale Shea Medical Center	Scottsdale	Arizona
Banner University Medical Center - Tucson	Tucson	Arizona
Arkansas Children's Hospital	Little Rock	Arkansas
KF - Orange County / Anaheim	Anaheim	California
Alta Bates Summit Medical Center	Berkeley	California
Providence St. Joseph Medical Center	Burbank	California
Mercy San Juan Medical Center	Carmichael	California
KF - Downey	Downey	California
KF - Fontana	Fontana	California
Fountain Valley Regional Hospital & Medical Center	Fountain Valley	California
Community Regional Med. Center - Fresno	Fresno	California
Saddleback Memorial Medical Center	Laguna Hills	California
Loma Linda University Children's Hospital	Loma Linda	California
Miller Children's and Women's Hospital at Long Bea	Long Beach	California
Adventist Health - White Memorial	Los Angeles	California
California Hospital Medical Center - Los Angeles	Los Angeles	California
Cedars-Sinai Medical Center (CSMC)	Los Angeles	California
KF - Sunset/Los Angeles	Los Angeles	California
LAC/USC (Los Angeles County, University Southern C	Los Angeles	California
UCLA - Mattel Children's Hospital at Ronald Reagan	Los Angeles	California
Northridge Hospital Medical Center	Northridge	California
KP - Oakland	Oakland	California
UC Irvine Medical Center	Orange	California
Desert Regional Medical Center	Palm Springs	California
Lucile Packard Children's Hospital Stanford	Palo Alto	California
Huntington Memorial Hospital	Pasadena	California
Pomona Valley Hospital Medical Center	Pomona	California
KP - Sacramento/Roseville	Roseville	California

© 2018 Ehret DEY et al. *JAMA Networ Open*.

Anderson Lucchetti Women's and Children's Center	Sacramento	California
UC Davis Medical Center	Sacramento	California
St. Bernadine Medical Center	San Bernardino	California
KF - San Diego	San Diego	California
Sharp Mary Birch Hospital for Women	San Diego	California
UCSD Medical Center - Hillcrest	San Diego	California
California Pacific Medical Center - West (CPMC)	San Francisco	California
KP - San Francisco	San Francisco	California
UCSF Benioff Children's Hospital in San Francisco	San Francisco	California
Good Samaritan Hospital, San Jose	San Jose	California
Santa Clara Valley Medical Center (SCVMC)	San Jose	California
Santa Barbara Cottage Hospital	Santa Barbara	California
KP - Santa Clara	Santa Clara	California
Santa Monica - UCLA Medical Center & Orthopaedic H	Santa Monica	California
St. John's Health Center	Santa Monica	California
Olive View - UCLA Medical Center	Sylmar	California
Providence Tarzana Medical Center	Tarzana	California
Harbor UCLA Medical Center	Torrance	California
Providence Little Company of Mary Medical Center	Torrance	California
Torrance Memorial Medical Center	Torrance	California
San Antonio Regional Hospital	Upland	California
Valley Presbyterian Hospital	Van Nuys	California
Community Memorial Hospital of Ventura	Ventura	California
Ventura County Medical Center (VCMC)	Ventura	California
John Muir Medical Center	Walnut Creek	California
Citrus Valley Medical Center	West Covina	California
Presbyterian Intercommunity Hospital (PIH Health)	Whittier	California
UCHSC	Aurora	Colorado
St. Francis Medical Center	Colorado Springs	Colorado
Denver Health Medical Center	Denver	Colorado
Rocky Mountain Hospital for Children at P/SL	Denver	Colorado
Saint Joseph Hospital	Denver	Colorado
St. Mary's Hospital and Medical Center	Grand Junction	Colorado
Connecticut Children's NICU at UCONN Health Center	Farmington	Connecticut
Connecticut Children's Medical Center	Hartford	Connecticut
St. Francis Hospital	Hartford	Connecticut
Yale-New Haven Children's Hospital	New Haven	Connecticut
Christiana Care Health Services	Newark	Delaware
MedStar Georgetown University Hospital	Washington	District Of Columbia

© 2018 Ehret DEY et al. *JAMA Networ Open*.

Brandon Regional Hospital	Brandon	Florida
Broward Health Medical Center/Salah Foundation Chi	Fort Lauderdale	Florida
Golisano Children's Hospital of Southwest Florida	Fort Myers	Florida
UF Shands Hospital Gainesville	Gainesville	Florida
Joe DiMaggio Children's Hospital	Hollywood	Florida
UF Health Jacksonville	Jacksonville	Florida
Wolfson Children's Hospital	Jacksonville	Florida
Baptist Children's Hospital	Miami	Florida
Jackson Memorial Hospital	Miami	Florida
Kendall Regional Medical Center	Miami	Florida
Florida Hospital for Children	Orlando	Florida
Winnie Palmer Hospital for Women and Babies	Orlando	Florida
Gulf Coast Regional Medical Center	Panama City	Florida
Sacred Heart Health System	Pensacola	Florida
Sheridan Children's, Plantation Hospital	Plantation	Florida
Tallahassee Memorial Hospital	Tallahassee	Florida
St. Joseph's Children's Hospital	Tampa	Florida
Tampa General Hospital	Tampa	Florida
Women's Center at Florida Hospital - Tampa	Tampa	Florida
St. Mary's Hospital	West Palm Beach	Florida
Emory University Hospital, Midtown	Atlanta	Georgia
Grady Memorial Hospital	Atlanta	Georgia
Northside Hospital	Atlanta	Georgia
Piedmont Hospital	Atlanta	Georgia
Augusta University Health System	Augusta	Georgia
Wellstar Cobb Hospital	Austell	Georgia
Medical Center at Columbus Regional, The	Columbus	Georgia
Memorial Health Savannah	Savannah	Georgia
Kaiser Permanente Moanalua Medical Center	Honolulu	Hawaii
Kapiolani Medical Center for Women & Children	Honolulu	Hawaii
St. Luke's Regional Medical Center	Boise	Idaho
Northwest Community Hospital	Arlington Heights	Illinois
Rush Copley Medical Center	Aurora	Illinois
Advocate Illinois Masonic Medical Center	Chicago	Illinois
John H. Stroger, Jr. Hospital of Cook County	Chicago	Illinois
Mt. Sinai Hospital Medical Center	Chicago	Illinois
Northwestern Memorial	Chicago	Illinois
Rush University Medical Center	Chicago	Illinois
St. Joseph Hospital Chicago	Chicago	Illinois
University of Chicago	Chicago	Illinois
University of Illinois at Chicago	Chicago	Illinois
Advocate Good Samaritan Hospital	Downers	Illinois

© 2018 Ehret DEY et al. *JAMA Networ Open*.

	Grove	
Evanston Hospital	Evanston	Illinois
Adventist Hinsdale Hospital	Hinsdale	Illinois
St. Alexius Medical Center	Hoffman Estates	Illinois
RMCH at Loyola University Medical Center	Maywood	Illinois
Edward Hospital and Health Services	Naperville	Illinois
Advocate Children's Hospital-Oak Lawn	Oak Lawn	Illinois
Advocate Children's Hospital - Park Ridge	Park Ridge	Illinois
CHOI at OSF St. Francis Medical Center	Peoria	Illinois
Rockford Memorial Hospital	Rockford	Illinois
St. John's Hospital	Springfield	Illinois
Carle Foundation Hospital	Urbana	Illinois
Central DuPage Hospital	Winfield	Illinois
IU Health North Hospital	Carmel	Indiana
St. Vincent Evansville	Evansville	Indiana
Dupont Hospital	Fort Wayne	Indiana
Lutheran Hospital of Indiana	Fort Wayne	Indiana
Parkview Women's and Children's Hospital	Fort Wayne	Indiana
Community Hospital North Indianapolis	Indianapolis	Indiana
Methodist Hospital of Indiana	Indianapolis	Indiana
Riley Hospital for Children at IU Health	Indianapolis	Indiana
St. Vincent Women's Hospital	Indianapolis	Indiana
Franciscan Health Lafayette	Lafayette	Indiana
Memorial Hospital	South Bend	Indiana
St. Luke's Hospital	Cedar Rapids	Iowa
Blank Children's Hospital	Des Moines	Iowa
Mercy Medical Center	Des Moines	Iowa
University of Iowa Children's Hospital	Iowa City	Iowa
University of Kansas Hospital Authority	Kansas City	Kansas
Overland Park Regional Medical Center	Overland Park	Kansas
Via Christi Hospitals Wichita	Wichita	Kansas
Wesley Medical Center	Wichita	Kansas
Kentucky Children's Hospital	Lexington	Kentucky
Norton Children's Hospital	Louisville	Kentucky
Christus St. Frances Cabrini Hospital	Alexandria	Louisiana
Rapides Women's and Children's Hospital	Alexandria	Louisiana
Baton Rouge General Medical Ctr - Bluebonnet	Baton Rouge	Louisiana
Woman's Hospital	Baton Rouge	Louisiana
Lafayette General Medical Center	Lafayette	Louisiana
Women's and Children's Hospital	Lafayette	Louisiana
Christus Lake Area Hospital	Lake Charles	Louisiana
Lake Charles Memorial Hosp for Women	Lake Charles	Louisiana
West Jefferson Medical Center	Marrero	Louisiana
Tulane Lakeside Hospital for Women and Children	Metairie	Louisiana

© 2018 Ehret DEY et al. *JAMA Networ Open*.

St. Francis Medical Center	Monroe	Louisiana
Ochsner Baptist Medical Center	New Orleans	Louisiana
Ochsner Medical Center	New Orleans	Louisiana
CHRISTUS Highland	Shreveport	Louisiana
LSU Health Sciences Center	Shreveport	Louisiana
Willis Knighton South	Shreveport	Louisiana
Eastern Maine Medical Center	Bangor	Maine
Barbara Bush Children's at Maine Medical	Portland	Maine
Anne Arundel Medical Center	Annapolis	Maryland
Franklin Square Hospital Center	Baltimore	Maryland
Greater Baltimore Medical Center	Baltimore	Maryland
JHBMC Hopkins Bayview Medical Ctr	Baltimore	Maryland
Johns Hopkins Hospital	Baltimore	Maryland
Sinai Hospital of Baltimore	Baltimore	Maryland
Univ of Maryland Medical Center	Baltimore	Maryland
Shady Grove Adventist Hospital	Rockville	Maryland
Holy Cross Hospital	Silver Spring	Maryland
Univ of Maryland St. Joseph Medical Ctr.	Towson	Maryland
Beth Israel Deaconess Medical Center	Boston	Massachusetts
Boston Medical Center	Boston	Massachusetts
Brigham and Women's Hospital	Boston	Massachusetts
Massachusetts General Hospital for Children	Boston	Massachusetts
Tufts Medical Center	Boston	Massachusetts
Baystate Medical Center	Springfield	Massachusetts
UMass Memorial Healthcare	Worcester	Massachusetts
St. Joseph Mercy Hospital	Ann Arbor	Michigan
U. of MI, CS Mott Children's, Brandon NICU	Ann Arbor	Michigan
Beaumont Hospital - Dearborn	Dearborn	Michigan
Henry Ford Hospital	Detroit	Michigan
Hutzel Women's Hospital	Detroit	Michigan
St. John Hospital & Medical Center	Detroit	Michigan
Hurley Medical Center	Flint	Michigan
DeVos Children's, Spectrum Health	Grand Rapids	Michigan
Children's Hospital at Bronson	Kalamazoo	Michigan
Sparrow Hospital	Lansing	Michigan
St. Joseph Mercy Oakland	Pontiac	Michigan
William Beaumont Hospital	Royal Oak	Michigan
Covenant Healthcare	Saginaw	Michigan
Essentia Health- St. Mary's Children's Hospital	Duluth	Minnesota
Children's Hospitals and Clinics- Minneapolis	Minneapolis	Minnesota
Hennepin County Medical Center	Minneapolis	Minnesota
University of MN Masonic Children's Hospital	Minneapolis	Minnesota
North Memorial Medical Center	Robbinsdale	Minnesota
Mayo Foundation	Rochester	Minnesota
St. Cloud Hospital	Saint Cloud	Minnesota

© 2018 Ehret DEY et al. *JAMA Networ Open*.

Children's Hospitals and Clinics- St Paul	Saint Paul	Minnesota
Forrest General Hospital	Hattiesburg	Mississippi
Children's Hospital at U. of MS Health Care	Jackson	Mississippi
Mississippi Baptist Health Systems	Jackson	Mississippi
North Mississippi Medical Center	Tupelo	Mississippi
St. Francis Medical Center	Cape Girardeau	Missouri
Women's & Children's Hosp, U. of MO	Columbia	Missouri
Freeman Hospital and Health System	Joplin	Missouri
Children's Mercy Kansas City	Kansas City	Missouri
Research Medical Center	Kansas City	Missouri
St. Luke's Hospital	Kansas City	Missouri
Truman Medical Center	Kansas City	Missouri
Mercy Children's Hospital , St. Louis	Saint Louis	Missouri
SSM Health Cardinal Glennon Children's Hospital	Saint Louis	Missouri
St. Louis Children's Hospital	Saint Louis	Missouri
Cox Health Neonatology	Springfield	Missouri
Mercy Kids Springfield	Springfield	Missouri
St. Vincent Hospital & Health Center	Billings	Montana
Benefis Healthcare	Great Falls	Montana
Community Medical Center	Missoula	Montana
St. Elizabeth Regional Medical Center	Lincoln	Nebraska
Creighton University Medical Center	Omaha	Nebraska
Methodist Women's Hospital	Omaha	Nebraska
Nebraska Medical Center	Omaha	Nebraska
St. Rose Dominican Hospital Siena Campus	Henderson	Nevada
Children's Hospital of Nevada at UMC	Las Vegas	Nevada
Sunrise Hospital and Medical Center	Las Vegas	Nevada
Dartmouth Hitchcock Medical Center	Lebanon	New Hampshire
Elliot Hospital	Manchester	New Hampshire
Children's at Cooper University Medical Center	Camden	New Jersey
Our Lady of Lourdes Medical Center	Camden	New Jersey
Hackensack University Medical Center	Hackensack	New Jersey
Saint Barnabas Medical Center	Livingston	New Jersey
Monmouth Medical Center	Long Branch	New Jersey
Goryeb Children's Hospital	Morristown	New Jersey
Jersey Shore University Medical Center	Neptune	New Jersey
Robert Wood Johnson University Hospital	New Brunswick	New Jersey
St. Peter's Medical Center	New Brunswick	New Jersey
Children's Hospital of New Jersey at NBIMC	Newark	New Jersey
University Hospital Rutgers-NJMS	Newark	New Jersey
St. Joseph Hospital and Medical Center	Paterson	New Jersey
Capital Health Medical Center-Hopewell	Pennington	New Jersey

© 2018 Ehret DEY et al. *JAMA Networ Open*.

Overlook Medical Center	Summit	New Jersey
Virtua Hospital Systems	Voorhees	New Jersey
Children's Medical Center at Presbyterian Hospital	Albuquerque	New Mexico
UNM School of Medicine Dept. of Pediatrics	Albuquerque	New Mexico
Albany Medical Center	Albany	New York
St. Peter's Hospital	Albany	New York
Jacobi Medical Center	Bronx	New York
Weiler Hospital Montefiore	Bronx	New York
Brookdale Hospital Medical Center	Brooklyn	New York
Brooklyn Hospital Center, The	Brooklyn	New York
Kings County Hospital Center	Brooklyn	New York
Maimonides Medical Center	Brooklyn	New York
University Hospital of Brooklyn	Brooklyn	New York
Sisters of Charity Hospital	Buffalo	New York
Arnot Ogden Medical Center	Elmira	New York
Queens Hospital Center	Jamaica	New York
North Shore University Hospital	Manhasset	New York
NYU Winthrop	Mineola	New York
Cohen Children's Medical Center of New York	New Hyde Park	New York
Bellevue Hospital	New York	New York
Columbia University Medical Center	New York	New York
Lenox Hill Hospital	New York	New York
Mt. Sinai Kravis Children's Hospital, The	New York	New York
Tisch Hospital, NYU Medical Center	New York	New York
Weill Cornell Medical Center	New York	New York
Vassar Brothers Hospital	Poughkeepsie	New York
Golisano Children's Hospital at Strong	Rochester	New York
Richmond University Medical Center	Staten Island	New York
Staten Island University Hospital	Staten Island	New York
Stony Brook University Medical Center	Stony Brook	New York
Good Samaritan Hospital Medical Center	West Islip	New York
Mission Children's Hospital	Asheville	North Carolina
North Carolina Children's Hospital	Chapel Hill	North Carolina
Carolinas Medical Center	Charlotte	North Carolina
Novant Health Presbyterian Medical Center	Charlotte	North Carolina
Jeff Gordon Children's Hospital at CMC	Concord	North Carolina
Duke University	Durham	North Carolina
Cape Fear Valley Medical Center	Fayetteville	North Carolina
Vidant Medical Center	Greenville	North Carolina
WakeMedical Center	Raleigh	North Carolina
NHRMC-Betty H.Cameron Women & Children's Hospital	Wilmington	North Carolina
Brenner Children's Hospital at WFUBMC	Winston-Salem	North Carolina

© 2018 Ehret DEY et al. *JAMA Networ Open*.

Forsyth Memorial Hospital	Winston-Salem	North Carolina
Sanford Bismarck Medical Center	Bismarck	North Dakota
Essentia Health	Fargo	North Dakota
Sanford Medical Center Fargo	Fargo	North Dakota
Altru Health System	Grand Forks	North Dakota
Children's Hospital Medical Center Cincinnati	Cincinnati	Ohio
Cleveland Clinic Foundation, The	Cleveland	Ohio
Fairview Hospital	Cleveland	Ohio
MetroHealth Medical Center	Cleveland	Ohio
Rainbow Babies & Children's Hospital	Cleveland	Ohio
Miami Valley Hospital	Dayton	Ohio
CCF Children's - Hillcrest NICU	Mayfield Heights	Ohio
Mercy Children's Hospital	Toledo	Ohio
Toledo Children's Hospital	Toledo	Ohio
O.U. Health Sciences Center	Oklahoma City	Oklahoma
Henry Zarrow Neonatal Intensive Care Unit	Tulsa	Oklahoma
Peggy V. Helmerich Women's Center	Tulsa	Oklahoma
St. John Medical Center	Tulsa	Oklahoma
St. Charles Health Care	Bend	Oregon
Oregon Health and Science University	Portland	Oregon
Providence St. Vincent Medical Center	Portland	Oregon
Randall Children's Hospital at Legacy Emanuel	Portland	Oregon
Sacred Heart Medical Center	Springfield	Oregon
Abington Memorial Hospital	Abington	Pennsylvania
Lehigh Valley Health Network	Allentown	Pennsylvania
St. Luke's University Hospital	Bethlehem	Pennsylvania
Bryn Mawr Hospital	Bryn Mawr	Pennsylvania
Geisinger Medical Center	Danville	Pennsylvania
Pinnacle Health Hospitals	Harrisburg	Pennsylvania
Penn State Children's Hospital	Hershey	Pennsylvania
Conemaugh Memorial Medical Center	Johnstown	Pennsylvania
Lancaster General Health-Women & Babies Hospital	Lancaster	Pennsylvania
Holy Redeemer Hospital and Medical Center	Meadowbrook	Pennsylvania
Children's Hospital of Philadelphia Newborn Center	Philadelphia	Pennsylvania
Einstein Medical Center Philadelphia	Philadelphia	Pennsylvania
Hospital of the University of Pennsylvania	Philadelphia	Pennsylvania
Pennsylvania Hospital	Philadelphia	Pennsylvania
Temple University Hospital	Philadelphia	Pennsylvania
Thomas Jefferson University Hospital	Philadelphia	Pennsylvania
Western Pennsylvania Hospital	Pittsburgh	Pennsylvania
Reading Hospital-Tower Health	Reading	Pennsylvania

© 2018 Ehret DEY et al. *JAMA Networ Open*.

Crozer Chester Medical Center	Upland	Pennsylvania
CHOP Newborn Care at Chester County Hospital	West Chester	Pennsylvania
Lankenau Medical Center	Wynnewood	Pennsylvania
WellSpan York Hospital	York	Pennsylvania
University Pediatric Hospital	San Juan	Puerto Rico
Women & Infants Hospital	Providence	Rhode Island
Medical University of South Carolina	Charleston	South Carolina
Palmetto Health Baptist,Women's & Neonatal Svcs	Columbia	South Carolina
Palmetto Health Richland	Columbia	South Carolina
McLeod Regional Medical Center	Florence	South Carolina
Children's Hospital of Greenville	Greenville	South Carolina
Spartanburg Regional Healthcare System	Spartanburg	South Carolina
Rapid City Regional Hospital	Rapid City	South Dakota
Avera McKennan	Sioux Falls	South Dakota
Boekelheide NICU at Sanford Health	Sioux Falls	South Dakota
Children's Hospital at Erlanger	Chattanooga	Tennessee
Niswonger Children's Hospital	Johnson City	Tennessee
University of Tennessee Medical Center	Knoxville	Tennessee
Baptist Memorial Hospital for Women	Memphis	Tennessee
Children's Hospital at TriStar Centennial, The	Nashville	Tennessee
Monroe Carell Jr. Children's Hospital Vanderbilt	Nashville	Tennessee
Baptist St. Anthony's Health System	Amarillo	Texas
Texas Tech University Health Science Center	Amarillo	Texas
Texas Health Arlington Memorial Hospital	Arlington	Texas
North Austin Medical Center	Austin	Texas
Seton Medical Center	Austin	Texas
St. David's Medical Center	Austin	Texas
CHRISTUS Southeast Texas Hospital	Beaumont	Texas
Valley Regional Medical Center TX	Brownsville	Texas
Christus Spohn Hospital Corpus Christi South	Corpus Christi	Texas
Corpus Christi Medical Center	Corpus Christi	Texas
Baylor Healthcare System	Dallas	Texas
Medical City Dallas	Dallas	Texas
Methodist Dallas Medical Center	Dallas	Texas
Presbyterian Hospital of Dallas	Dallas	Texas
Texas Health Presbyterian Hospital Dallas	Dallas	Texas
University of Texas Southwestern Med. Ctr. Dallas	Dallas	Texas
William P. Clements Jr. University Hospital	Dallas	Texas
Women's Hospital At Renaissance	Edinburg	Texas
Del Sol Medical Center	El Paso	Texas
Las Palmas Medical Center	El Paso	Texas
Baylor All Saints Medical Center	Fort Worth	Texas
Cook Children's Medical Center	Fort Worth	Texas
Harris Methodist Fort Worth Hospital	Fort Worth	Texas
University of Texas Medical Branch	Galveston	Texas

© 2018 Ehret DEY et al. *JAMA Networ Open*.

Ben Taub General Hospital	Houston	Texas
Children's Memorial Hermann Hospital	Houston	Texas
Lyndon B. Johnson General Hospital	Houston	Texas
Memorial Hermann Southwest	Houston	Texas
St. Joseph Hospital	Houston	Texas
Texas Children's Hospital, Baylor College of Med.	Houston	Texas
Woman's Hospital of Texas, The	Houston	Texas
Medical City Lewisville	Lewisville	Texas
Covenant Women and Children's Hospital	Lubbock	Texas
Medical City Plano	Plano	Texas
Texas Health Presbyterian Hospital Plano	Plano	Texas
Children's Hospital of San Antonio	San Antonio	Texas
Methodist Children's Hospital	San Antonio	Texas
North Central Baptist Hospital	San Antonio	Texas
St. Luke's Baptist Hospital	San Antonio	Texas
University Hospital San Antonio	San Antonio	Texas
McLane Children's Hospital	Temple	Texas
Clear Lake Regional Medical Center	Webster	Texas
McKay Dee Hospital Center	Ogden	Utah
Utah Valley Regional Medical Center	Provo	Utah
Dixie Regional Medical Center	Saint George	Utah
St. Mark's Hospital	Salt Lake City	Utah
University of Utah Health Sciences Center	Salt Lake City	Utah
University of Vermont Children's Hospital	Burlington	Vermont
University of Virginia	Charlottesville	Virginia
INOVA Children's Hospital	Falls Church	Virginia
Children's Hospital of the King's Daughters	Norfolk	Virginia
Bon Secours Richmond Health System	Richmond	Virginia
Children's Hosp of Richmond at VCU	Richmond	Virginia
CJW Medical Center, Chippenham Campus	Richmond	Virginia
Henrico Doctors' Hospital	Richmond	Virginia
Carilion Clinic Children's Hospital	Roanoke	Virginia
Evergreen Health	Kirkland	Washington
Swedish Medical Center	Seattle	Washington
University of Washington Medical Center	Seattle	Washington
Deaconess Hospital	Spokane	Washington
Providence Sacred Heart Medical Center & Childrens	Spokane	Washington
MultiCare Health System-Tacoma General Hospital	Tacoma	Washington
Charleston Area Medical Center	Charleston	West Virginia
Cabell Huntington Hospital, Inc.	Huntington	West Virginia
West Virginia University School of Medicine	Morgantown	West Virginia
Aurora Baycare Medical Center	Green Bay	Wisconsin
St. Vincent Hospital	Green Bay	Wisconsin
Gundersen Lutheran Medical Center	La Crosse	Wisconsin

© 2018 Ehret DEY et al. *JAMA Networ Open*.

Meriter Hospital	Madison	Wisconsin
SSM Health St. Mary's Hospital Medical Center	Madison	Wisconsin
St. Joseph Hospital-Marshfield Clinic	Marshfield	Wisconsin
Ascension - St. Joseph's Hospital	Milwaukee	Wisconsin
Aurora Sinai Medical Center	Milwaukee	Wisconsin
Children's Hospital of Wisconsin	Milwaukee	Wisconsin
Columbia St. Mary's Hospital	Milwaukee	Wisconsin
Children's Hospital of Wisconsin, Fox Valley	Neenah	Wisconsin
Waukesha Memorial Hospital	Waukesha	Wisconsin
Aspirus Wausau Hospital	Wausau	Wisconsin

© 2018 Ehret DEY et al. *JAMA Networ Open*.
